# Study on the Regulatory Mechanism of Niacin Combined with *B. animalis F1-7* in Alleviating Alcoholic Fatty Liver Disease by Up-Regulating GPR109A

**DOI:** 10.3390/nu16234170

**Published:** 2024-11-30

**Authors:** Xi Liang, Tianhu Shan, Xiumei Zheng, Zhe Zhang, Yanping Fan, Huaqi Zhang, Lanwei Zhang, Hui Liang

**Affiliations:** 1 Department of Nutrition and Food Hygiene, School of Public Health, Qingdao University, 308 Ningxia Road, Qingdao 266071, China; liangxi6029@163.com (X.L.); tianhu0427@163.com (T.S.); zxm2569954642@163.com (X.Z.); 18369613510@163.com (Y.F.) huaqi_erin_qy@qdu.edu.cn (H.Z.); 2 College of Food Science and Engineering, Ocean University of China, Qingdao 266003, China; zhangzhe_hmu@126.com

**Keywords:** alcoholic fatty liver, niacin, probiotics, lipid metabolism, gut microbiota

## Abstract

Background: This study aimed to investigate the effects of niacin combined with *B. animalis* F1-7 on the improvement of alcoholic fatty liver disease (AFLD) in mice and its potential regulatory mechanism. Methods: A total of 75 8-week-old male C57BL/6N mice were acclimated for one week and randomly divided into five groups: control group, alcohol model group (AFLD), niacin intervention group (NA), *B. animalis* F1-7 intervention group (F1-7), and niacin combined with *B. animalis* F1-7 intervention group (NF). The experiment lasted for 8 weeks. Results: The results showed that all intervention groups could effectively reduce the serum lipid levels and inflammatory response of mice induced by alcohol to varying degrees. The immunofluorescence analysis showed that the GPR109A in the liver and intestine of the NF group was significantly enhanced compared with the other groups. Niacin combined with *B. animalis* F1-7 better restored the gut microbiota. Meanwhile, each intervention group could increase their levels of SCFAs. Among them, the combination group increased the levels of acetic acid and butyric acid more significantly than the other two groups. The Spearman’s correlation analysis of gut microbiota and SCFAs showed that *Norank_f_Eubacterium_coprostanoligenes_group*, *Allobaculum*, and *Akkermansia* were positively correlated with changes in SCFAs, while *Coriobacteriaceae_UCG-002*, *Romboutsia*, and *Clostridium_sensu_stricro_1* were negatively correlated. Conclusions: Niacin combined with *B. animalis* F1-7 better regulated the gut microbial balance and increased the SCFAs in mice with alcoholic steatohepatitis. The mechanism was related to the activation of the target GPR109A, which regulates the key proteins involved in lipid synthesis and *β*-oxidation to improve lipid metabolic disorders.

## 1. Introduction

Alcohol consumption can cause health damage, especially liver injury [1]. Studies have shown that most patients with liver disease have imbalances in their gut microbiota [2]. Alcohol destroys the intestinal barrier and gut microbial balance, causing the migration of harmful substances such as endotoxin, producing large amounts of inflammatory factors, and aggravating liver damage [3]. Therefore, paying attention to intestinal health and the gut microbial balance is crucial for alcoholic liver disease.

Niacin is an essential vitamin for the human body. Long-term drinking can lead to the deficiency of niacin, and it is necessary to supplement niacin in moderation [4]. Niacin has good lipid-lowering function and has been used clinically as a lipid-lowering drug for many years [5]. However, high doses of niacin can cause a flushing reaction, so the intervention intake of niacin should be kept within a safe range [6]. Niacin acts on the G protein-coupled receptor 109A (GPR109A) to regulate lipid metabolism [7]. Studies have shown that SCFAs can also act on this receptor and play a regulatory role [8]. This provides a new method for reducing the dosage of niacin and maintaining physiological function.

Probiotics play an important role in the intestinal microbiota. They can participate in human digestion and absorption, promote intestinal peristalsis and produce a variety of metabolites [9]. Probiotic supplementation is capable of rehabilitating a damaged intestinal barrier and augmenting the levels of SCFAs, thereby attenuating liver injury [10]. The organic acids produced by common probiotics such as *Bifidobacterium* and Lactobacillus can regulate intestinal pH and prevent the growth of pathogenic bacteria [11]. The *Bifidobacterium animalis* subsp. *lactis* F1-7 (*B. animalis* F1-7) utilized in this study is a probiotic strain renowned for its anti-inflammatory and lipid-lowering attributes, as well as its regulatory role on the intestinal microbiota [12,13]. Previous studies have shown that B vitamins combined with probiotics are good for one’s health [14]. However, the mechanism is still poorly understood, with information regarding that of niacin combined with probiotics on alcoholic liver injury being especially scarce. It is of interest to not only reduce the dose of niacin but also increase its protective effect against AFLD by adding probiotics.

This research endeavors to explore the protective effects of niacin and *B. animalis* F1-7 on alcoholic fatty liver disease in mice. The AFLD mouse model was established through alcohol feeding, and interventions with niacin and *B. animalis* F1-7 were administered. Commencing from the key target GPR109A, this study evaluated alterations in the intestinal microbiota and the ameliorative effects of combined intervention on AFLD so as to provide more safe and effective improvement methods for AFLD.

## 2. Materials and Methods

### 2.1. Strain and Culture

The strain used in this experiment was *B. animalis* F1-7 (no. CCTCC M 2020833), which was stored in the Department of Nutrition and Food Hygiene. The *B. animalis* F1-7 was inoculated into an MRS medium and cultured at 37 °C for 48 h. After, the strains were activated twice and centrifuged, and the supernatant was removed to obtain a bacterial precipitate, which was subsequently washed twice more with a sterile phosphate buffer (PBS). The total viable count was adjusted to 1 × 10^9^ CFU/mL for later use.

### 2.2. Animal Model and Sample Collection

We purchased 75 SPF male C57BL/6N mice aged 8 weeks (20 ± 2 g) from Beijing Vital River Laboratory. The mice were housed individually in a room with constant conditions: temperature (21–25 °C), humidity (50–60%), and a 12 h light/dark cycle. After one week of adaptation, the mice were randomly divided into 5 groups, each consisting of 15 mice: the control group (control), alcohol-induced liver disease model group (AFLD), niacin intervention group (NA), *B. animalis* F1-7 intervention group (F1-7), and niacin and *B. animalis* F1-7 mixed intervention group (NF). The control group was fed a 4% Lieber–DeCarli liquid diet, while the other four groups received a 4% Lieber–DeCarli liquid alcohol diet. The mice in the control and AFLD groups were administered PBS at a dose of 5 mL/kg/d by gavage. The NA group received niacin at a dose of 50 mg/kg/d by gavage. Group F1-7 was given a bacterial solution containing 1 × 10^9^ CFU/mL by gavage. The NF group received both niacin at a dose of 50 mg/kg and bacterial solution containing 1 × 10^9^ CFU/mL by gavage. The intervention experiment lasted for 8 weeks.

On the sixth day of the 9th week, fresh fecal samples were collected frozen with liquid nitrogen. The last day, all mice underwent a 12 h fasting period and were then anesthetized via an intraperitoneal injection of sodium pentobarbital and extracted to obtain blood samples. The obtained serum was subjected to centrifugation for biochemical indicator analysis. The livers and small intestines of the mice were collected, some of which were fixed in 4% paraformaldehyde for histopathological observation samples and some of which were stored in a refrigerator at −80 °C for follow-up index detection.

### 2.3. Determination of Body Weight and Liver Index

The body weight of mice in each group was recorded weekly throughout the experiment. Livers were removed immediately after mice were sacrificed and weighed. The liver index was calculated using the formula: liver index (%) = liver weight (g)/body weight (g).

### 2.4. Detection of Serum Biochemical Indicators

The levels of alanine aminotransferase (ALT), aspartate aminotransferase (AST), triglyceride (TG), total cholesterol (TC), low-density lipoprotein cholesterol (LDL-C) and high-density lipoprotein cholesterol (HDL-C) in serum were measured with an automatic biochemical analyzer.

### 2.5. Measurement of TG and TC in Liver

The TG and TC levels of the liver were measured with kits (Nanjing Jiancheng Bioengineering Institute, Nanjing, China).

### 2.6. Serum Inflammatory Factor Detection

The serum levels of endotoxin (LPS), tumor necrosis factor-α (TNF-α), interleu-kin-1β (IL-1β) and interleukin-6 (IL-6) were detected with enzyme-linked immuno-sorbent assay (ELISA) kits according to the manufacturer’s instructions (Jianglai Bio-Technology Co., Ltd., Shanghai, China).

### 2.7. Histological Analysis

Mouse liver and small intestine tissues were fixed in 4% paraformaldehyde, embedded in paraffin and cut into 5 μm slices. Paraffin sections of the liver were stained with H&E and Oil Red O [15]. Paraffin sections of small intestinal tissue were treated with H&E staining.

### 2.8. Immunofluorescence and Immunohistochemical Staining

The liver and small intestine sections were processed for immunofluorescence staining using GPR109A [16]. The immunohistochemical staining of intestinal tight junction proteins was performed using the following primary antibodies: ZO-1, Occludin, and Claudin-1 (Abcam, Cambridge, MA, USA). The images were observed under a light microscope (Olympus, Center Valley, PA, USA) and analyzed with Image J software 1.54g [13].

### 2.9. Determination of Key Protein Expression by Western Blotting

The total protein content of the mouse liver tissue was lysed and extracted with a high-efficiency RIPA lysate and enzyme inhibitor mixture. Proteins with adjusted concentrations were added to the loading wells of prepared SDS-PAGE gels (8–10%) for electrophoresis. After protein separation, the proteins were transferred to a PVDF membrane. The primary antibodies included ACC, p-ACC, SREBP1, CPT1A, FAS, and β-actin (diluted at 1:1000, Abcam, Cambridge, MA, USA). After incubation in goat anti-rabbit IgG–HRP secondary antibody (1:7500, Cell Signaling, Danvers, MA, USA), the protein bands were detected with a high-sensitivity ECL solution (Beyotime, Haimen, China), and the gray values were analyzed with Image J software 1.54g [17].

### 2.10. Sequencing Analysis of 16srRNA

Mouse feces were sent to Shanghai Meiji Biomedical Technology Co., Ltd. (Shanghai, China) for 16S rRNA gene sequencing, and the total DNA of the samples was extracted, amplified and sequenced according to its standard procedures. The universal primers for 338F: 5′-ACTCCTACGGGAGGCAGCAG-3′ and 806R: 5′-GGACTACHVGGGTWTCTAAT-3′—were selected to amplify the V3–V4 region. The sequencing library was established and sequenced on the Illumina HiSeq platform to obtain data information. The statistical data analysis was completed on the platform (https://www.majorbio.com) (accessed on 15 March 2024) [18].

### 2.11. Measurement of Intestinal Short-Chain Fatty Acids

The determination of intestinal short-chain fatty acids was performed with gas chromatography by mixing the intestinal contents. Samples with 1200 μL of distilled water were homogenized by continuous vortexing for 1 min. The suspension was added to 50 mL of 50% sulfuric acid and vortexed for 5 min. Subsequently, the samples were centrifuged at 5000× *g* for 10 min. The supernatant was transferred to a 1.5 mL tube, and 50 μL of the internal standard (2-ethyl butyrate solution) and 300 μL of anhydrous diethyl ether were added. The tubes were turned upside down for 30 s and centrifuged at 5000× *g* for 10 min. Then, 1 μL of the upper diethyl ether layer was injected into the chromatograph injection port for analysis. A DB-FFAP (30 m × 0.25 mm × 0.25 μm) capillary column was used to separate SCFAs [19].

### 2.12. Statistical Analysis

SPSS 22.0 software was used for statistical analysis, and the analysis results were expressed as mean ± standard deviation (SD). A one-way analysis of variance (ANOVA) was used to compare the quantitative data between multiple groups, and the Tukey method was used to compare the differences between groups. Different letters indicate significant differences. *p* < 0.05 was considered statistically significant. GraphPad Prism 8.0 was used for mapping.

## 3. Results

### 3.1. Effects of Niacin and B. animalis F1-7 on Body Weight and Liver Index

There was no significant difference in food intake among the groups of mice in the experiment (Supplementary Materials). Alcohol can lead to decreased liver function, affecting the body’s metabolism and nutrient absorption ability, resulting in weight loss. The results showed that almost all the mice fed with the alcohol-containing liquid diet showed weight loss, with no significant difference between groups (Table 1). In addition, Table 1 shows that the liver index of the AFLD group was significantly higher than that of the control (*p* < 0.05). The three intervention groups showed decreased liver index values to varying degrees compared with the AFLD group (*p* < 0.05). Compared with the F1-7 group, the liver index of the NF group decreased more significantly (*p* < 0.05).

### 3.2. Effects of Niacin and B. animalis F1-7 on Liver Injury

**Figure 1:**
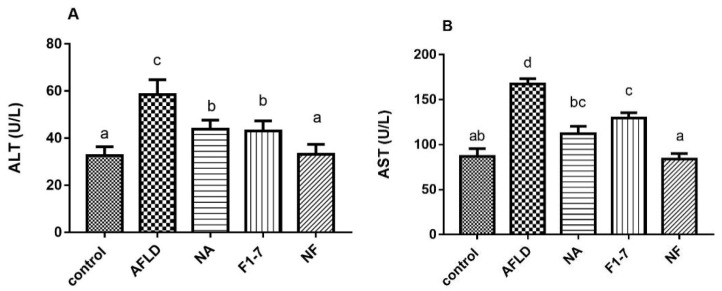
Effects of niacin and *B. animalis* F1-7 on serum markers of liver injury in mice. (**A**) Serum ALT; (**B**) serum AST. The difference between histograms marked by different letters was significant, *p* < 0.05.

Alcohol intervention increased the level of serum ALT; compared with the AFLD group, and the NA, F1-7 and NF groups had significant reductions in serum ALT. The effect of the combined intervention was significantly better than that of the other two groups alone, and ALT in the combined intervention group was decreased by 41% compared with the model group. Meanwhile, the niacin, F1-7, and combination groups ameliorated the alcohol-induced abnormal elevation of AST levels to varying degrees (Figure 1A). AST levels were down-regulated by 31% in the NA group and by 20% in the F1-7 group compared with the AFLD group. The combination of the two interventions was most effective, with a 38% reduction (Figure 1B).

### 3.3. Effects of Niacin and B. animalis F1-7 on Lipid Disorders

**Figure 2:**
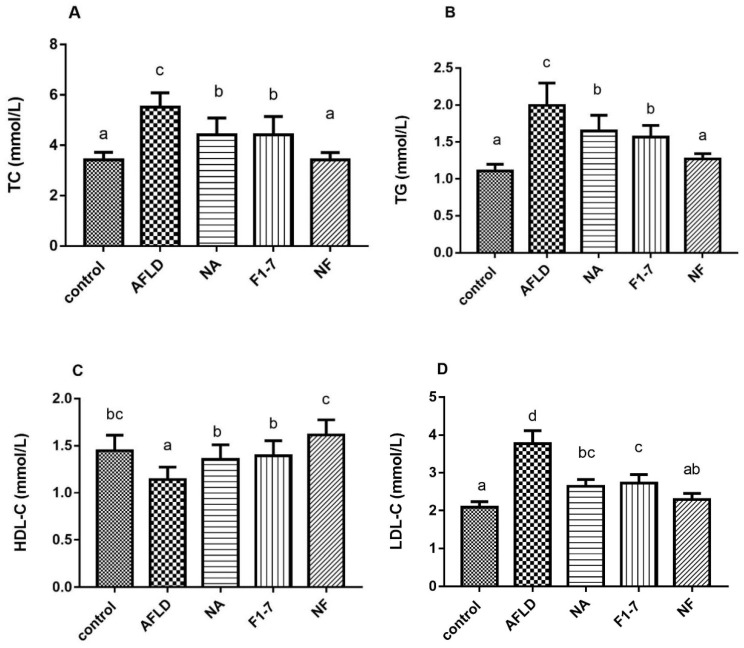
Effects of niacin and *B. animalis* F1-7 on lipid metabolism in mice. (**A**) Serum TC; (**B**) serum TG; (**C**) serum HDL-C; (**D**) serum LDL-C. The difference of histograms marked by different letters was significant, *p* < 0.05.

The TC level in the AFLD group was significantly higher than that in the control group, and the probiotic, niacin, and combination interventions effectively reduced the serum TC content (*p* < 0.05). Compared with the model group, the TC levels of the NA and F1-7 groups decreased by 20% and 21%. That of the NF group decreased by 39%, which was significantly lower than the other two groups (*p* < 0.05). The change trend of TG after intervention was consistent with that of TC. The NF group was significantly more effective than the other two intervention groups, reducing the TG level by 38% and showing no significant difference with the control group (Figure 2B). The alcohol treatment reduced the serum HDL-C levels in the mice (Figure 2C). All intervention groups showed increased HDL-C levels, with the combined intervention having the greatest effect with an increase of 42%. The NA, F1-7 and NF interventions effectively reduced the level of LDL-C (Figure 2D) by 31%, 29%, and 40%, respectively, compared with the model group. Serum indicators indicated that the niacin combined with probiotics intervention effectively improved alcohol-induced serum lipid disorders, and the effect was better than either of them alone.

### 3.4. Effects of Niacin and B. animalis F1-7 Intervention on Serum Inflammatory Factor

**Figure 3:**
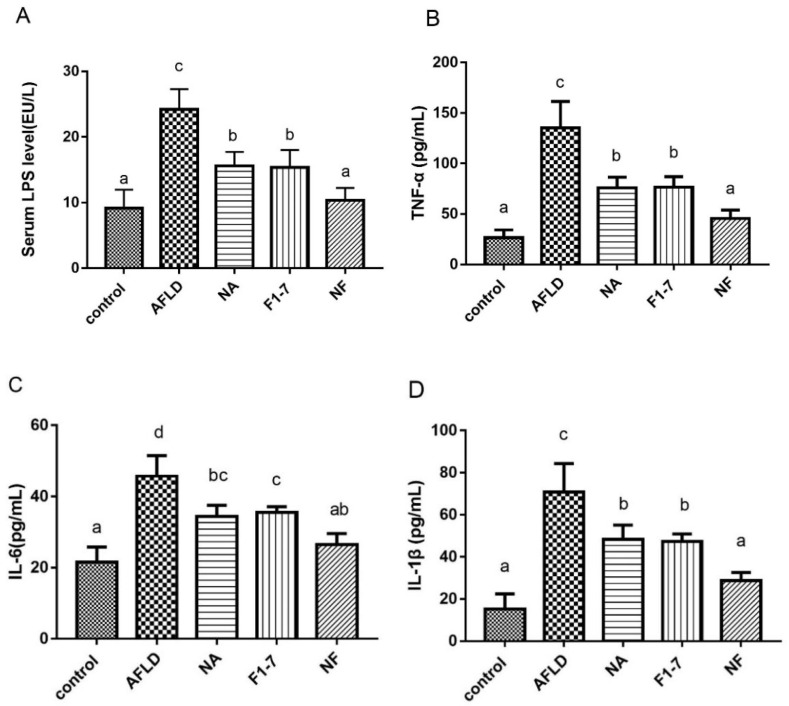
Effects of niacin and *B. animalis* F1-7 on serum levels of inflammatory factors in mice. (**A**) Serum LPS; (**B**) serum TNF-α; (**C**) serum IL-6; (**D**) serum IL-1β. The difference of histograms marked by different letters was significant, *p* < 0.05.

The alcohol intervention significantly increased serum LPS levels, while the niacin and probiotics interventions were effective in improving serum LPS levels (Figure 3A). Compared with the AFLD group, the niacin group was down-regulated by 36% and the F1-7 group was down-regulated by 37%. The effect of the mixed intervention was significantly better than the other two groups (*p* < 0.05), as the effect was down-regulated by 57%. The serum levels of TNF-α were significantly different in the alcohol intervention group and the control group (*p* < 0.05) (Figure 3B). The intervention group had a better effect than the other two groups, with an effect that was reduced by 66% compared with the model group. Alcohol significantly increased the serum level of IL-6 in the mice (*p* < 0.05), niacin reduced it by 25%, and F1-7 reduced it by 22%. The effect of the NF group was better than that of the probiotic group. The serum IL-1β levels were basically consistent with those of TNF-α (Figure 3D). Both the NA and F1-7 intervention groups could effectively reduce IL-1β levels, and the NF group had the best effect. Niacin combined with *B. animalis* F1-7 could improve the inflammatory response in the mice, and the effect was better in combination than either alone.

### 3.5. Effects of Niacin and B. animalis F1-7 on Liver Lipids

**Figure 4:**
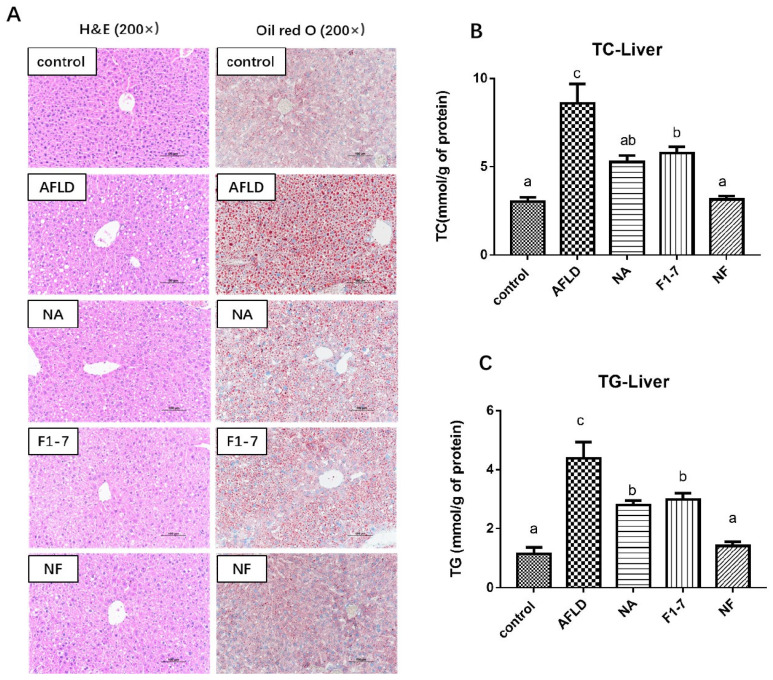
Histopathological changes and measures of liver lipid levels in mice. (**A**) H&E and Oil Red O staining (Scale bar: 100 μm); (**B**) liver TC; (**C**) liver TG. The difference of histograms marked by different letters was significant, *p* < 0.05.

Compared with control group, the hepatocytes of the AFLD group showed diffuse fatty lesions, more different sizes of vesicular steatosis, and a large number of inflammatory cells infiltration in the hepatic lobule. The three intervention groups had significantly less fatty liver lesions, fewer lipid droplets, and less inflammatory cell infiltration than the AFLD group (Figure 4A). The alcohol intervention significantly increased the content of TC in the liver of the mice (*p* < 0.05). Compared with the AFLD group, TC levels were down-regulated by 39% in the NA group and by 33% in the F1-7 group, with no significant difference. The NF group had a 63% reduction in liver TC content, which was relatively more effective than the other groups (Figure 4B). The TG levels were significantly increased in the AFLD mice, while the niacin and F1-7 interventions reduced the liver TG content by 36% and 32%, respectively (Figure 4C). The NF group had a 68% reduction in liver TG. The combination group had the best effect.

### 3.6. Effects of Niacin and B. animalis F1-7 on Intestinal Barrier Key Protein Expression

**Figure 5:**
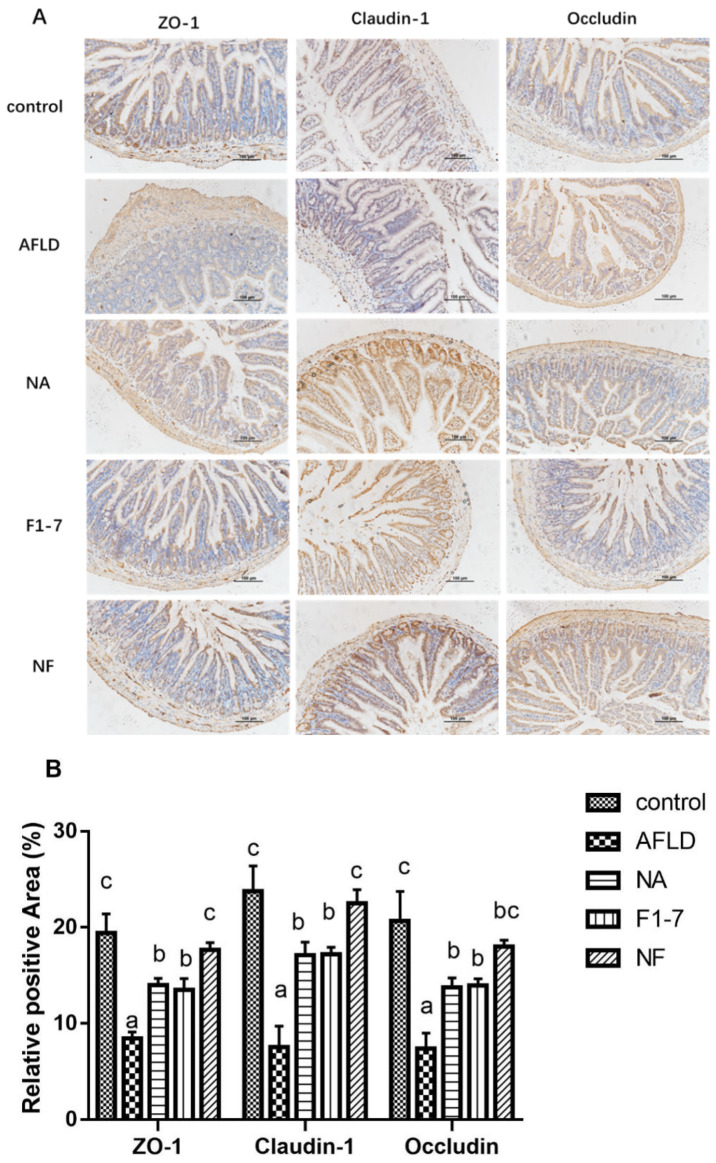
(**A**) Immunohistochemical staining of the key proteins for mouse intestinal barrier (Scale bar: 100 μm); (**B**) quantitative analysis of the positive area of ZO-1, Claudin-1 and Occludin. Different letters indicate significant differences, *p* < 0.05.

The analysis of the intestinal barrier of mice showed that the expression of the ZO-1 protein was significantly down-regulated in the model group compared with the control group (*p* < 0.05) according to immunohistochemistry (Figure 5A). The niacin, F1-7 and combination interventions effectively increased the content of ZO-1 in intestinal cells, and the content of ZO-1 in the NF group was significantly higher than that in the NA and F1-7 groups (*p* < 0.05). Compared with the control group, the positive area of Claudin-1 in the model group was significantly down-regulated (Figure 5B). The three intervention groups could up-regulate Claudin-1 to different degrees and increase the positive area of Claudin-1. The combined effect of niacin and probiotics was better than that of niacin and probiotics alone (*p* < 0.05). The alcohol intervention reduced Occludin expression. Compared with the model group, the expression of Occludin in the intestinal tracts of the mice treated with niacin and probiotics was significantly increased (*p* < 0.05). The results demonstrated that the niacin combined with probiotics intervention could effectively improve the intestinal barrier.

### 3.7. Effects of Niacin and B. animalis F1-7 on the Expression of GPR109A

**Figure 6:**
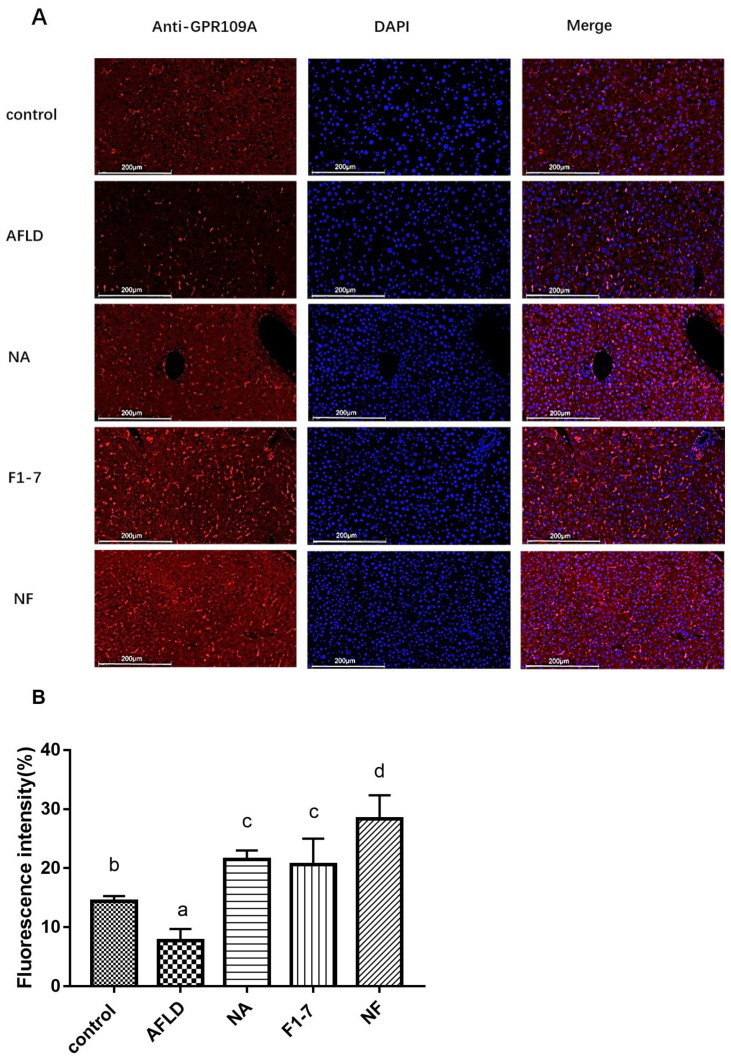
Immunofluorescence staining (**A**) and fluorescence intensity (**B**) of GPR109A in liver tissue. Different letters indicate significant differences, *p* < 0.05.

**Figure 7:**
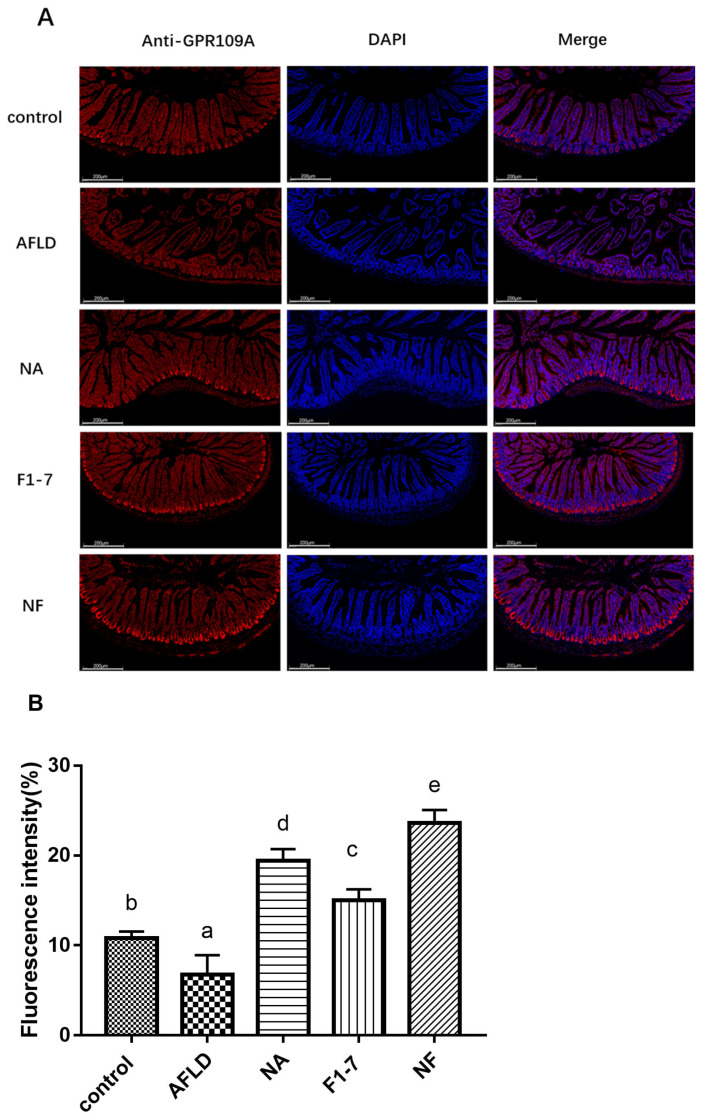
Immunofluorescence staining (**A**) and fluorescence intensity (**B**) of GPR109A in the small intestinal tissue. Different letters indicate significant differences, *p* < 0.05.

The immunofluorescence analysis of GPR109A in the liver showed that alcohol intervention significantly reduced the expression of GPR109A (Figure 6). The level of GPR109A in the liver of the mice in the combined niacin and probiotics intervention group was significantly increased, and this effect was better than that in the niacin and *B. animalis* F1-7 groups alone. The intestinal fluorescence immunohistochemistry of GPR109A was further analyzed. The alcohol model group showed a lower fluorescence intensity of GPR109A than the control group. The niacin, *B. animalis* F1-7, and combined interventions effectively increased the expression of GPR109A in small intestinal tissue, and the niacin group was more effective than the probiotics intervention group (*p* < 0.05). The combined intervention group had the best effect (Figure 7).

### 3.8. Effects of Niacin and B. animalis F1-7 on the Expression of Proteins Related to Lipid Metabolism in the Liver

**Figure 8:**
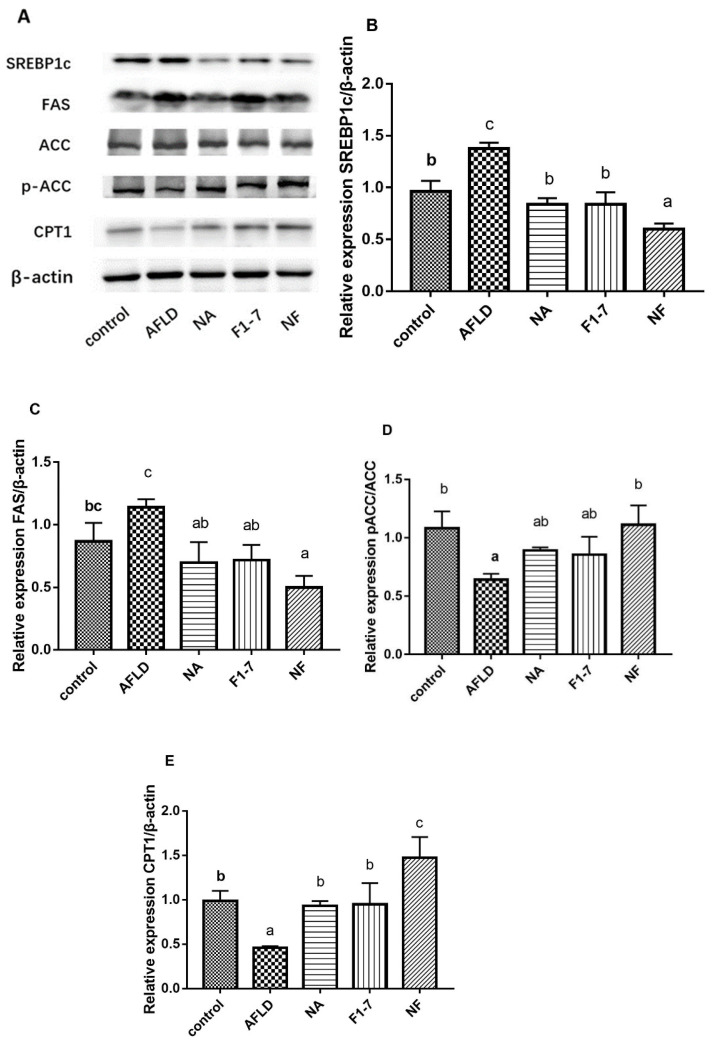
(**A**) Mouse liver protein expression results of Western blotting; (**B**) the relative expression level of SREBP1c liver protein; (**C**) the relative expression level of FAS liver protein; (**D**) the ratio of the relative expression levels of p-ACC and ACC liver proteins; (**E**) the relative expression level of CPT1 protein in liver. Different letters indicate significant differences, *p* < 0.05.

Figure 8 shows that compared with the control group, the SREBP1c protein content in the AFLD group increased by 43%. The niacin, *B. animalis* F1-7 and mixed interventions reduced its expression (*p* < 0.05). The NF group had the best effect and was down-regulated by 56%. The alcohol induction increased the expression of FAS in the mouse livers by 32% compared with the control group. Its expression was reduced by 39% after niacin intervention and by 37% in the F1-7 group. The NF group had a 56% reduction compared with the AFLD group. The results showed that alcohol could reduce the phosphorylation level of ACC, and niacin combined with *B. animalis* F1-7 increased the expression of p-ACC. The p-ACC/ACC ratio was significantly decreased in the model mice compared with the AFLD mice, with reductions of 31%, 32% and 50% in the NA, F1-7, and NF groups, respectively. Meanwhile, the expression of key lipid catabolism protein CPT1 was significantly decreased by 54% (*p* < 0.05) after alcohol treatment. The niacin and F1-7 groups effectively increased its expression. Compared with the AFLD group, the combined treatment of niacin and probiotics increased the protein expression of CPT1 by 2.2 fold. These results indicate that the alcohol intervention increased hepatic lipid synthesis and promoted lipid accumulation.

### 3.9. Effects of Niacin and B. animalis F1-7 on Gut Microbiota

**Figure 9:**
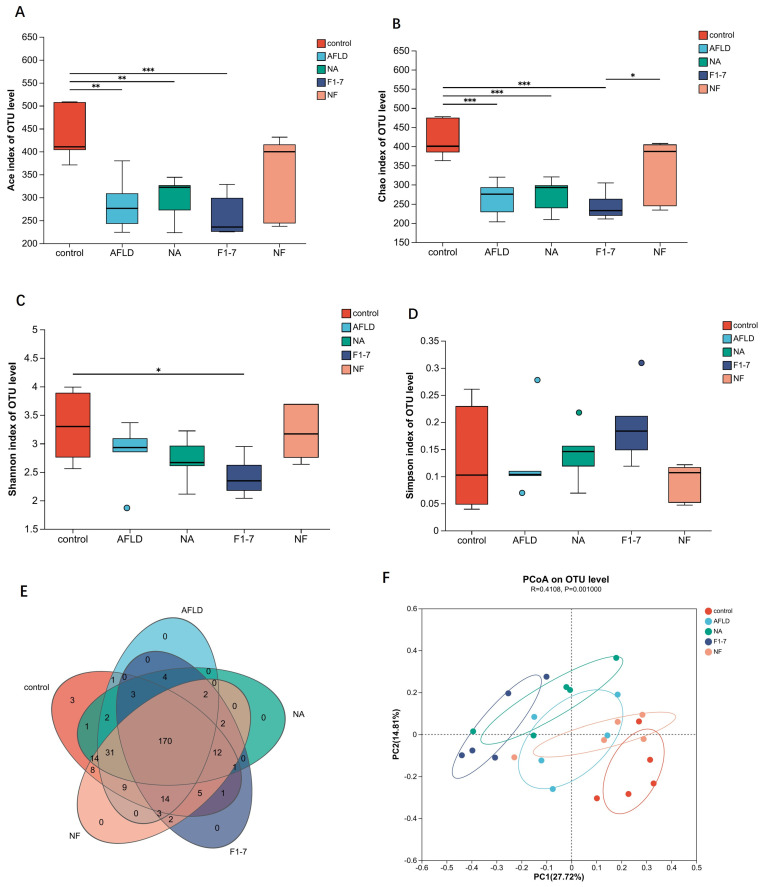
Effects of niacin and *B. animalis* F1-7 on gut microbiota of mice; (**A**) ACE index; (**B**) Chao index; (**C**) Shannon index; (**D**) Simpson index; (**E**) Venn diagram; (**F**) principal coordinate analysis (PCoA). * *p* < 0.05, ** *p* < 0.01, *** *p* < 0.001.

We analyzed the gut microbiota with 16S rRNA sequencing, and the Venn plots showed that 170 OTUs were identified in the feces of each group (Figure 9E). According to ACE, Shannon and Chao index analyses, the AFLD group had a significant reduction in the richness of gut microbiota, and the NF group had a significant increase in the richness of gut microbiota (Figure 9A–D). The alpha diversity of the gut microbiota in the NF group was significantly better than that in the AFLD group and the other two intervention groups. The principal coordinate analysis (PCoA) analysis showed the relative distribution characteristics of beta diversity (Figure 9F). The results showed that there were significant differences between the control group and the model group, as well as significant differences between the intervention groups and the model group.

**Figure 10:**
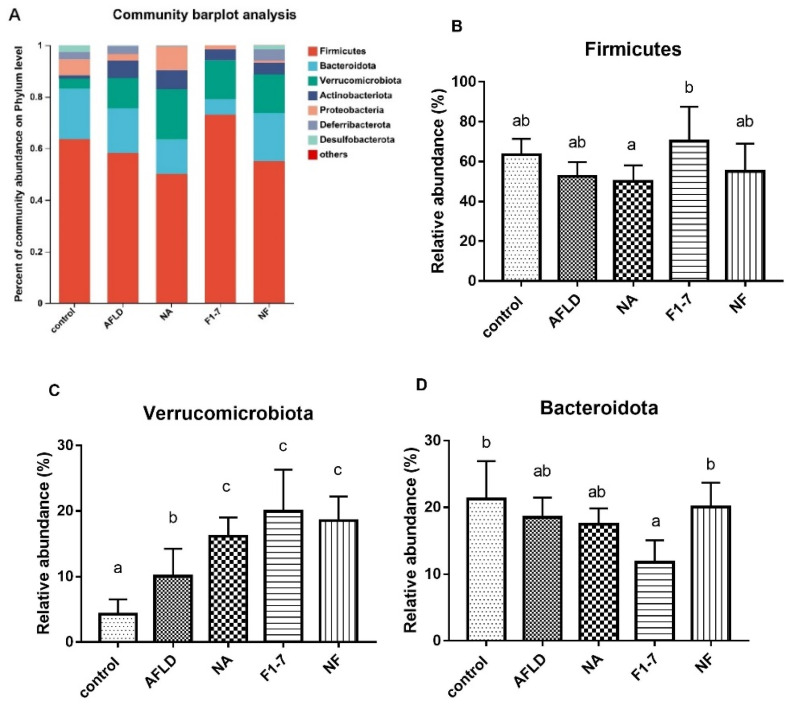

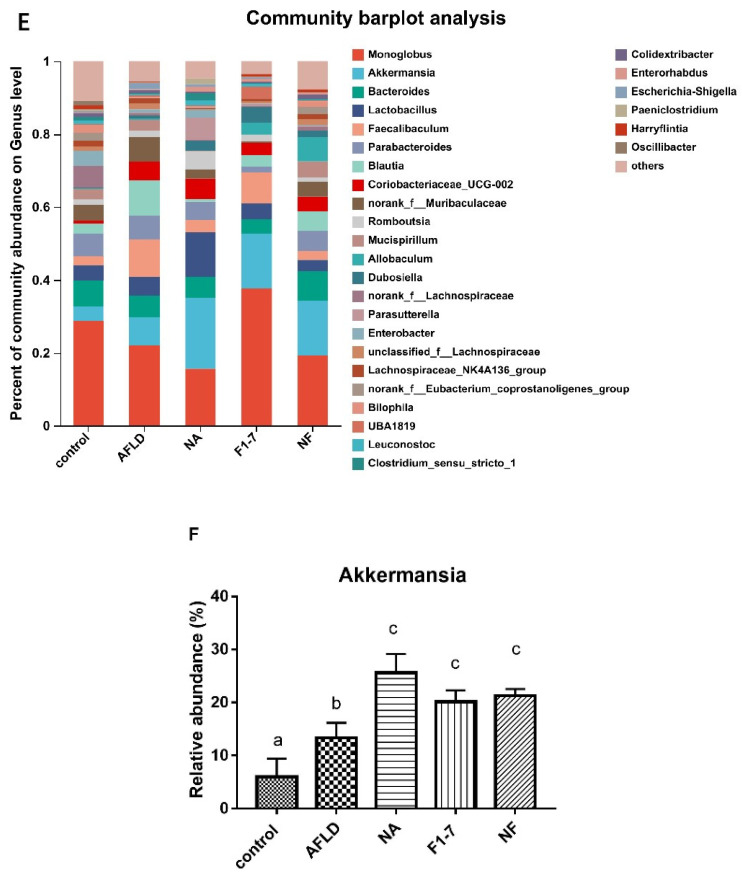
Effects of niacin and *B. animalis* F1-7 on gut microbiota in mice. (**A**) Bar chart of community structure at phylum level; (**B**) the relative abundance of Firmicutes; (**C**) the relative abundance of Verrucomicrobiota; (**D**) the relative abundance of Bacteroidota; (**E**) analysis of community structure at genus level; (**F**) the relative abundance of *Akkermansia*. Different letters indicate significant differences, *p* < 0.05.

The gut microbiota in each group were composed of Firmicutes, Bacteroidota, Verrucomicrobiota, Actinobacteriota, Proteobacteria and Desferriobacteriota at the phylum level (Figure 10A). Firmicutes and Bacteroidota were dominant (Figure 10B,D). In addition, there were significant changes in the abundance of Verrucomicrobia (Figure 10C). Compared with the model group, the intervention groups could significantly up-regulate the abundance of Verrucomicrobia. The further analysis of the microbiota structure at the genus level revealed significant changes in *Akkermansia* in Verrucomicrobia (Figure 10E). Compared with the model group, the niacin, probiotics, and mixed groups could effectively increase the relative abundance of *Akkermansia* (Figure 10F).

**Figure 11:**
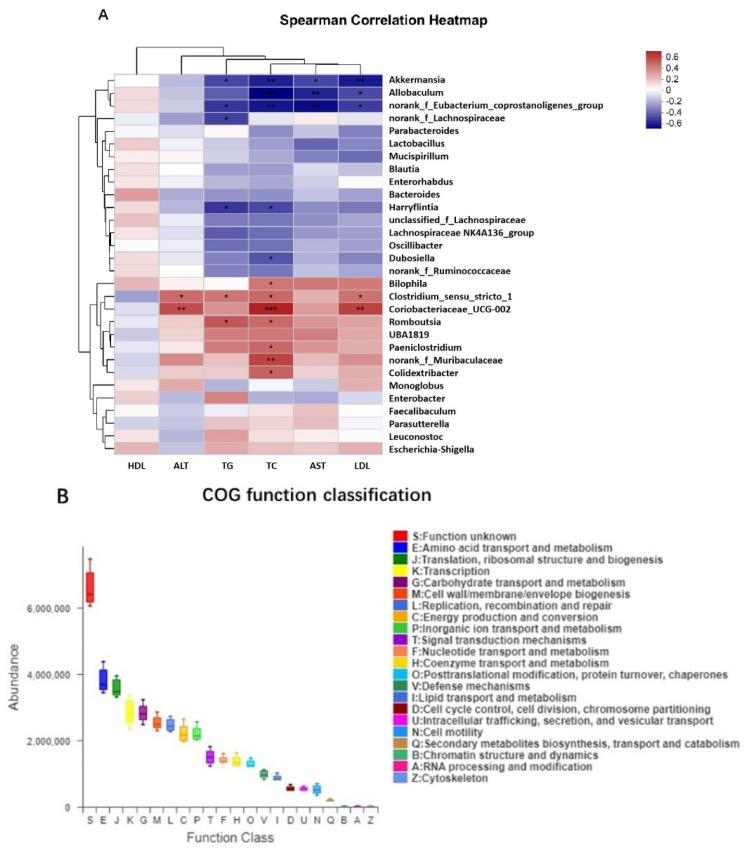
(**A**) Spearman correlation analysis heatmap; (**B**) lineal homologous group (COG) analysis. * *p* < 0.05, ** *p* < 0.01, *** *p* < 0.001.

The correlation between gut microbiota and key serum indicators at the genus level was analyzed (Figure 11A). *Coriobacteriaceae_UCG-002*, *Clostridium_sensu_stricto_1*, *Romboutsia*, *norank_f_Muribaculaceae*, *Bilophila*, *Paeniclostridiu* and *Colidextribacter* were positively correlated with serum TC. *Romboutsia* and *Clostridium_sensu_stricto_1* were positively correlated with serum TG. *Coriobacteriaceae_UCG-002* and *Clostridium_sensu_stricto_1* were positively correlated with serum LDL and ALT related to liver function. Among the changing gut microbiota, *Allobaculum* was negatively correlated with serum TC, LDL, and aspartate aminotransferase related to liver function. *Norank_f_Eubacterium_coprostanoligenes_ group* and *Akkermansia* were negatively correlated with multiple indicators in serum including TG, TC, LDL and AST. *Harryflintia* was negatively correlated with the serum lipids TG and TC. *Norank_f_Lachnospiraceae* was negatively correlated with TG, and *Dubosiella* was negatively correlated with TC. According to the COG functional distribution, the representative functional distribution included 21 functional categories such as amino acid transport and metabolism, cell wall/membrane/envelope biosynthesis, replication, recombination and repair, carbohydrate transport and metabolism, and lipid transport and metabolism (Figure 11B).

### 3.10. Effects of Niacin and B. animalis F1-7 on Intestinal Short-Chain Fatty Acids

**Figure 12:**
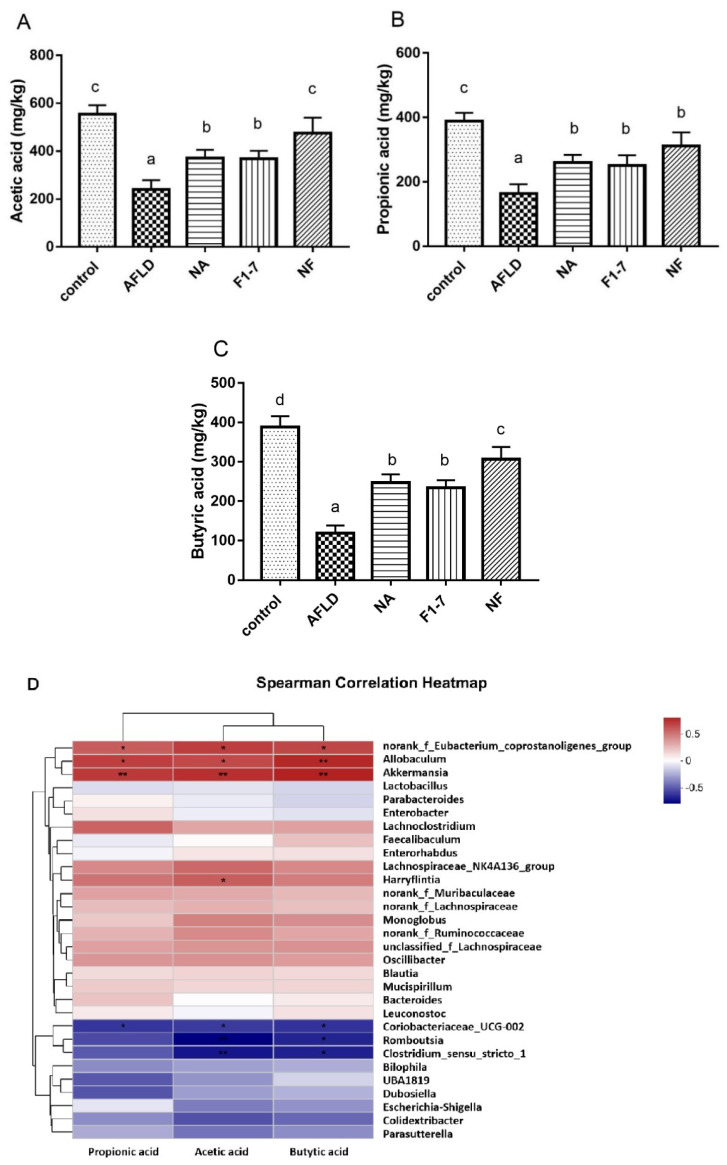
(**A**) Acetic acid level; (**B**) propionic acid level; (**C**) butyric acid level; (**D**) heatmap of Spearman correlation analysis. * *p* < 0.05, ** *p* < 0.01. The difference of histograms marked by different letters was significant, *p* < 0.05.

The contents of three SCFAs in intestinal contents were determined. Alcohol consumption resulted in a significant reduction of the intestinal metabolites acetate, propionate, and butyrate. All three intervention groups were able to increase the content of the intestinal metabolites SCFAs to varying degrees. The effect of the NF group on increasing acetic acid and butyric acid was significantly better than that of niacin or *B. animalis* F1-7 alone (*p* < 0.05) (Figure 12A–C). Spearman correlation analysis showed that *Allobaculum*, *norank_f_Eubacterium_coprostanoligenes_group* and *Akkermansia* were positively correlated with the changes of SCFAs. *Harryflintia* was positively correlated with acetic acid. *Romboutsia* and *Clostridium_sensu_stricro_1* were negatively correlated with acetic acid and butyrate. *Coriobacteriaceae_UCG-002* was negatively correlated with acetic acid, propionic acid, and butyric acid (Figure 12D).

## 4. Discussion

The human liver is the main organ for alcohol metabolism, and about 80% of drinkers have liver damage, which will gradually develop into hepatitis, liver fibrosis and even liver cancer [20]. Intestinal homeostasis is highly correlated with AFLD. Alcoholic liver disease is affected early by metabolites produced by the disturbance of microbiota. The inflammation and lipid metabolism caused by an impaired intestinal barrier can aggravate liver damage [21]. So, the early regulation of intestinal homeostasis is particularly important in delaying AFLD.

Body weight and the liver index were used to determine the overall nutritional status and liver abnormalities of mice exposed to long-term alcohol intake. In our study, it was found that the mice in the AFLD group showed a decreased body weight and an increased liver index, while the NA group, F1-7 group and NF group showed different degrees of decrease in the liver index. Both niacin and *B. animalis* F1-7 improved liver enlargement, AST and ALT in the mice exposed to long-term alcohol intake. In addition, there is also evidence that alcohol can also change lipid metabolism [22]. In this experiment, the levels of the serum lipid-related indexes TG, TC and LDL-C of the alcohol-exposed mice were significantly increased, while the levels of HDL-C were decreased, indicating that the model group had lipid metabolism disorders. Both niacin and *B. animalis* F1-7 reversed alcohol-induced liver injury and lipid-related serological abnormalities in the mice, and the combined intervention improved these indicators more significantly. The early stage of alcoholic liver injury is often characterized by hepatic steatosis, the accumulation of fat in liver cells. In this study, it was found that the liver tissue of mice in the AFLD group showed large and numerous fat vacuoles accompanied by inflammatory cell infiltration, and the levels of TG and TC in the liver were also significantly increased. The combination of niacin and *B. animalis* F1-7 better improved lipid accumulation and inflammatory cell infiltration, and the effect of the NF group on reducing liver TG and TC levels was more significant than that of the NA and F1-7 groups.

It has been suggested that intestinal barrier imbalance was an important factor in the development of alcoholic liver [23]. Our study showed that the positive area of ZO-1, Claudin-1 and Occludin related to intestinal tight junction protein decreased significantly in the AFLD group, while the improvement effect was more obvious in the mixed group. These results indicated that niacin combined with *B. animalis* F1-7 repaired the intestinal barrier damage caused by alcohol. Meanwhile, long-term alcohol stimulation increases intestinal permeability, which will lead the LPS to activate Kupffer cells and produce a large number of pro-inflammatory cytokines that cause inflammation and aggravate the development of alcoholic liver disease [24,25]. In this experiment, the serum LPS of AFLD mice increased significantly and the levels of TNF-α, IL-6 and IL-1β also increased significantly. Alcohol exposure caused inflammation in mice, and both niacin and probiotics significantly inhibited the levels of inflammatory factors, though the combination of interventions was better. Niacin and *B. animalis* F1-7 reduced liver injury by protecting the intestinal barrier.

Previous papers have pointed out that GPR109A, as a liver sensor, can play a protective role in liver fat accumulation [26]. GPR109A is involved in mediating the lipid-lowering effect of niacin, reducing lipid synthesis through the AMPK–ACC pathway, and inhibiting the mTOR signaling pathway [27]. GPR109A receptor activation can reduce cyclic adenosine phosphate (cAMP) production and protein kinase A (PKA) activity, reducing free fatty acid (FFA) release [28]. However, the excessive intake of niacin can cause some adverse reactions such as flushing. Studies have shown that a high daily intake of niacin (more than 1 g) increases the risk of adverse reactions [29]. In our study of the combination group, we reduced the dose of niacin but found that the regulatory effect of the combination intervention on GPR109A was enhanced. It was found with immunofluorescence staining that the combination intervention also enhanced the expression of the GPR109A receptor in the mouse liver tissue. In addition, the lipid metabolism genes in the liver were significantly regulated in the combination group. SREBP1c regulated key enzymes related to lipid synthesis, such as downstream FAS, and promoted liver adipogenesis by up-regulating their expression [30]. ACC was regulated by AMPK and SREBP1c [31] and promoted the conversion of malonyl-CoA, an inhibitor of CPT1, thereby inhibiting the progress of fatty acid β-oxidation [32]. In this study, we observed an alcohol-induced enhancement of SREBP1c and FAS expression in the mouse livers, which was reversed to varying degrees, and increased ACC phosphorylation and CPT1 levels. The combination of niacin and *B. animalis* F1-7 weakened the liver lipid synthesis and enhanced the function of fatty acid β oxidation.

Probiotics regulated the expression of GPR109A by regulating metabolite composition in the gut. The metabolites through which probiotics activated GPR109A need to be further investigated. Studies have shown that GPR109A is not only a niacin receptor but can also bind to SCFAs, especially butyric acid, to play a regulatory role [33]. In this experiment, the immunofluorescence results of GPR109A in the intestinal tract showed that the receptor was activated in both the niacin and probiotic groups. We further tested the intestinal short-chain fatty acid levels of the mice. Compared with the model group, the intestinal acetic acid, propionic acid and butyric acid levels in the three intervention groups were all increased, and the increases of the acetic acid and butyric acid levels in the NF group were more significant than those in the intervention group alone. These results suggest that niacin combined with *B. animalis* F1-7 had a remarkable effect on the content of intestinal metabolite butyric acid.

The structure and abundance of gut microbiota can affect the production of metabolites [34]. Therefore, we analyzed the gut microbiota in mice with 16S rRNA microbial diversity gene sequencing. Diversity analysis showed that the species richness and diversity of the intestinal microbes in the mice fed a liquid alcohol diet decreased compared with those in the control group, and obvious changes in gut microbiota composition of mice in the model group were observed with PCoA analysis. In general, the three intervention groups all showed different degrees of improvement. Among these interventions, the NF group played the most obvious regulatory role on the gut microbiota, and the richness and diversity of intestinal microorganisms increased and approached the levels of the control group. Our study further analyzed the relative abundance of the gut microbiota at the phylum and genus levels. Firmicutes and Bacteroidota showed higher relative abundances at the phylum level in this study. At the same time, we found that the combination intervention effectively increased the abundance of Verrucomicrobiota. Verrucomicrobiota is one of the common intestinal microorganisms in the human body that can reduce inflammation and participate in lipid and energy metabolism, playing a positive role in human health [35,36]. We further observed the gut microbiota levels and found that compared with the model group, the relative abundance of *Akkermansia* in Verrucomicrobiota was significantly improved in the three intervention groups. Studies have shown that changes in the abundance of *Akkermansia* are correlated with changes in the expression of lipid metabolism and inflammatory markers [37,38].

In order to further explore the relationship between alcoholic fatty liver disease and gut microbiota, we performed a Spearman correlation analysis on the serological indicators of liver injury and lipid metabolism, as well as the relative abundance of gut microbiota at the genus level. The results showed that a variety of bacteria, including *Akkermansia* and *Allobaculum*, were significantly negatively correlated with serum lipid levels and liver damage indexes in the mice. *Coriobacteriaceae_UCG-002* and *Clostridium_sensu_stricto_1* showed positive correlations with related serological indicators. This study found that *Allobaculum* was negatively correlated with the serum lipid levels of the mice, while *Coriobacteriaceae_UCG-002* was positively correlated, which was similar to the results of a previous study [39]. In addition, COG functional analysis suggested that the potential microbial functional characteristics of niacin combined with probiotic in the intestinal tract of mice with alcoholic liver injury may be related to lipid transport and metabolic signal transduction. The above research results show that the combination of niacin with F1-7 inhibited the structural abnormalities of the flora caused by alcohol, and its ability to regulate gut microbiota was significantly enhanced compared with niacin or F1-7 alone.

Changes in gut microbiota will directly affect the contents of intestinal SCFAs [40]. In our study, the three major SCFA levels in the AFLD group of mice were significantly decreased, and the combination group remarkably decreased SCFAs compared with individual groups. The correlation analysis between SCFAs and gut microbiota revealed that *Allobaculum*, *Akkermansia* and *norank_f_Eubacterium_coprostanoligenes*_group were positively correlated with the changes of the three main SCFAs. *Coriobacteriaceae_UCG-002*, *Romboutsia* and *Clostridium_sensu_stricro_1* were negatively correlated with SCFA levels. Studies have shown that *Allobaculum* has the ability to produce butyric acid [41] and *Akkermansia* can promote the production of SCFAs, especially butyric acid [42]. A Spearman’s correlation heat map showed a correlation between butyric acid levels and intestinal microbiota, indicating that the niacin and probiotics intervention may affect butyric acid through the regulation of the above bacteria in the intestine and then exert a more significant regulatory effect on activating the intestinal GPR109A target. In addition, except for SCFAs, other metabolites may also change after the combined intervention of niacin and probiotics, such as β-hydroxybutyric acid, another ligand of GPR109A. The effects of these differential metabolites on key targets and regulatory pathways still need to be further analyzed.

## 5. Conclusions

In summary, the supplementation of niacin combined with *B. animalis* F1-7 showed a better improvement effect on the lipid metabolism disorders and inflammatory injuries of alcoholic fatty liver compared with niacin alone. The combination group maintained the integrity of the intestinal barrier to reduce inflammatory injury. Meanwhile, the combination group improved the abnormal accumulation of fat by regulating the liver SREBP1c/FAS/ACC/CPT1 pathway. The combination group could better regulate the composition of SCFA-producing bacteria, thereby increasing the content of SCFAs and synergically increasing the expression of GPR109A. As vitamins and probiotics continue to be widely used, this study explores the synergies between their combined use for human health.

## Figures and Tables

**Table 1:** Effects of interventions on body weight and liver index of mice.

Group	Body Weight (g)	Liver Index (%)
Control	27.74 ± 1.28 ^b^	4.43 ± 0.26 ^a^
AFLD	22.53 ± 1.30 ^a^	5.80 ± 0.52 ^c^
NA	23.88 ± 1.38 ^a^	4.94 ± 0.17 ^a,b^
F1-7	23.04 ± 1.72 ^a^	5.21 ± 0.32 ^b^
NF	23.66 ± 1.98 ^a^	4.62 ± 0.22 ^a^

Note: Data with different letters were significantly different, *p* < 0.05.

## Data Availability

The datasets used and/or analyzed during the current study are available from the corresponding author upon reasonable request. The data are not publicly available due to [pricacy].

## Supplementary Materials

The following supporting information can be downloaded at: https://www.mdpi.com/article/10.3390/nu16234170/s1, Figure S1: There was no significant difference in food intake among the groups of mice in the experiment.

## Abbreviations

AFLD, alcoholic fatty liver disease; GPR109A, G protein-coupled receptor 109A; SCFAs, short-chain fatty acids; ALT, alanine aminotransferase; AST, aspartate aminotransferase; TC, total cholesterol; TG, total triglycerides; HDL-C, high-density lipoprotein; LDL-C, low-density lipoprotein; LPS, endotoxin; TNF-α, tumor necrosis factor-α; IL-1β, interleukin-1β; IL-6, interleukin-6; SREBP1c, sterol regulatory element-binding protein-1c; FAS, fatty acid synthase; ACC, acetyl-CoA carboxylase; CPT1, human carnitine palmitoyltransferase I.
