# Supplementary material for: Study on the Regulatory Mechanism of Niacin Combined with B. animalis F1-7 in Alleviating Alcoholic Fatty Liver Disease by Up-Regulating GPR109A
Source: Nutrients. 2024 Nov 30;16(23):4170. doi: 10.3390/nu16234170 (PMC11644097; doi:10.3390/nu16234170)

Figure S1: There was no significant difference in food intake among the groups of mice in the experiment.

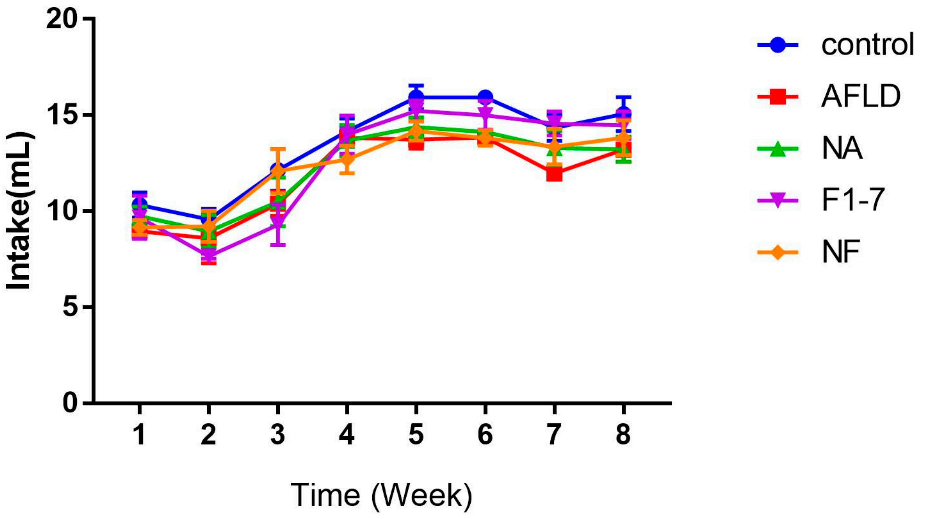

Supplement: Supplementary file 1 [file nutrients-16-04170-s001.zip › nutrients-3330026-supplementary.pdf]
